# Improving Responsiveness: Our Journey from Manual Yearly Updates to Automated Linkage for Near Real-Time Understanding of Outcomes and Modelling Future Service Demand

**DOI:** 10.23889/ijpds.v9i5.2516

**Published:** 2024-09-18

**Authors:** Philip Witowski, Mark Sipthorp, Adam Ismail, Windra Sulaiman, Beverley Phillips, Sharon Williams

**Affiliations:** 1 Centre for Victorian Data Linkage

## Objective

Demand for real-time data during the COVID-19 pandemic revealed a need to increase efficiencies in manual linkage processes to respond to events in near real-time. In response, our jurisdictional linkage agency transitioned from yearly to daily, weekly and monthly linkage practices through increasing automation and improving process flows.

## Approach

Our linkage agency transitioned to a fully automated process utilising scalable cloud infrastructure. Source data is now provided directly to a common data platform. This data is split into linkage and content, cleansed and quality assured in Python and set to automatically run via Azure data pipelines. The data is then linked via a combination of deterministic and probabilistic criteria, with data quality checks automatically performed along the way. Researchers can analyse this data in a secure virtual machine that only they can access and retrieve data from.

## Results

This infrastructure expedites the data linkage process allowing daily linkage results to select datasets, and enables advanced research such as a predictive micro-simulation model, which leverages the platform to predict and intervene on outcomes influenced by governmental policies. This model relies on timely administrative data to build targeted interventions for groups with poor future outcomes, tests these interventions, and monitors outcomes in near real-time.

## Conclusion

Our linkage agency has transitioned from manual to automated linkage processes in response to increasing need for timely data. By embracing cloud infrastructure and leveraging automation, we have streamlined our operations, enabling responsive linkage depending on need and expediting the provision of de-identified, linked data to researchers.

